# Association of therapeutic plasma exchange with functional outcomes in selected neurological disorders: a retrospective cohort study from a single tertiary center in Saudi Arabia

**DOI:** 10.3389/fneur.2026.1876331

**Published:** 2026-07-21

**Authors:** Fatemah I. Ammar, Esraa Aljahdali, Amal Alshrif, Asmaa Alshareef, Dania Faidah, Mohammed Almohammadi, Seraj Makkawi

**Affiliations:** 1 College of Medicine, King Saud Bin Abdulaziz University for Health Sciences, Jeddah, Saudi Arabia; 2 King Abdullah International Medical Research Center, Jeddah, Saudi Arabia; 3 Department of Hematology, Ministry of National Guard Health Affairs, Jeddah, Saudi Arabia; 4 Department of Neurology, Ministry of National Guard Health Affairs, Jeddah, Saudi Arabia

**Keywords:** autoimmune encephalitis, chronic inflammatory demyelinating polyneuropathy, Guillain-Barré syndrome, multiple sclerosis, myasthenia gravis, neuromyelitis optica spectrum disorder, therapeutic plasma exchange

## Abstract

**Background:**

Neurological disorders are associated with considerable morbidity and healthcare impact. Therapeutic plasma exchange (TPE) is an intervention that removes pathogenic antibodies, immune complexes, and other plasma constituents from circulation. Previous studies have suggested that TPE could be associated with improved outcomes in selected neurological disorders.

**Objectives:**

To evaluate functional outcomes following therapeutic plasma exchange in patients with selected neurological disorders at National Guard Health Affairs (NGHA) in Jeddah, Saudi Arabia.

**Methods:**

This retrospective cohort study analyzes 133 patients diagnosed with various neurological disorders who received TPE between August 2015 and December 2022 at NGHA. Diagnoses included multiple sclerosis (MS), myasthenia gravis (MG), Guillain-Barré syndrome (GBS), neuromyelitis optica spectrum disorder (NMOSD), chronic inflammatory demyelinating polyneuropathy (CIDP), autoimmune encephalitis (AE), and other neurological conditions including amyotrophic lateral sclerosis (ALS), transverse myelitis, cerebellar ataxia, paraneoplastic neurologic syndrome, polymyositis, and central nervous system (CNS) infections. The effectiveness of TPE was assessed using the Modified Rankin Scale (mRS) before and 90 days following the final TPE session. Statistical analysis included Wilcoxon signed-rank tests and Mann–Whitney U test, with significance set at (*p* < 0.05).

**Results:**

Significant improvements in mRS scores were observed following TPE for MS, MG, and GBS (all *p* < 0.001), as well as for NMOSD (*p* = 0.008), CIDP (*p* = 0.007), and AE (*p* = 0.018), but not in other neurological disorders (*p* = 0.234). Overall mortality was 6.8%, with no TPE-related deaths. Comorbidities among deceased patients included hypertension, diabetes mellitus, malignancy, chronic kidney disease, and cardiovascular disease.

**Conclusion:**

TPE was associated with improved functional outcomes in selected neurological disorders. Comorbidities were common among patients with poorer outcomes. Due to the retrospective design and potential confounding factors, causal inferences cannot be established. Further prospective studies are warranted to evaluate the role of TPE and optimize patient selection.

## 1 Introduction

Neurological disorders encompass a wide spectrum of conditions, including both autoimmune and non-autoimmune etiologies. Autoimmune neurological disorders occur when the immune system erroneously targets components of the nervous system, whereas non-autoimmune disorders may result from vascular, metabolic, infectious, or other pathological processes (1). Therapeutic plasma exchange (TPE) has been utilized as a treatment modality across this spectrum and is thought to exert its therapeutic effects through the removal of circulating pathogenic autoantibodies, immune complexes, and other plasma constituents that may contribute to disease pathology (1–3). Plasmapheresis refers to the separation and removal of plasma from whole blood (2). Therapeutic plasma exchange, in contrast, involves not only removal but also replacement of plasma with appropriate humoral components. Despite these technical distinctions, the literature often uses the terms interchangeably (2–7). The burden of immune-mediated neurological disorders, including myasthenia gravis (MG), autoimmune encephalitis (AE), Guillain–Barré syndrome (GBS), multiple sclerosis (MS), and neuromyelitis optica spectrum disorder (NMOSD), has increased globally and regionally (8–10).

To guide clinical decision-making, standardized recommendations have been established for the use of TPE across various neurological conditions. According to the American Society for Apheresis (ASFA) 2023 guidelines, TPE indications are categorized into four groups: Category I includes first-line indications such as GBS, MG, NMDA receptor encephalitis, and chronic inflammatory demyelinating polyneuropathy (CIDP). Category II encompasses conditions like acute disseminated encephalomyelitis, MS, and NMOSD, where TPE may serve as a second-line option (11). Category III includes diseases like crescentic IgA nephropathy, where the role of TPE is uncertain and should be individualized. In Category IV, TPE is generally not recommended due to lack of benefit or potential harm, as in thrombotic microangiopathy (11, 12).

While TPE is widely accepted as a therapeutic option (5), some literature has raised concerns regarding its safety and complications. Afzali et al. (7) highlighted the need for caution, particularly with central vascular access. Reported complications include allergic reactions, fluid shifts, cardiovascular instability, and infections related to vascular access. Hypocalcemia is among the most common adverse effects due to citrate anticoagulants used in the apheresis circuit (6, 8). In addition, intravenous immunoglobulin (IVIG) is used as a first-line or adjunctive therapy in several immune-mediated neurological disorders. Previous studies have reported comparable efficacy between IVIG and TPE in selected conditions (13, 14). For example, in Guillain–Barré syndrome, both IVIG and TPE are recommended treatment options according to the current European Academy of Neurology/Peripheral Nerve Society (EAN/PNS) guidelines. However, the role and effectiveness of these therapies vary across neurological disorders depending on disease characteristics, severity, and clinical context (15). Given the limited regional data and the clinical variability in outcomes, further investigation is warranted. Therefore, this study aims to assess the functional outcomes associated with TPE in patients with selected neurological disorders at NGHA in Jeddah, Saudi Arabia.

## 2 Materials and methods

### 2.1 Ethical approval

Before the start of this study, we received the IRB approval for the proposal and ethical consideration from King Abdullah International Medical Research Center (KAIMRC) with the reference number: JED-23-427780-54022. Also, the confidentiality of all patients’ information was assured.

### 2.2 Study population

This retrospective study included 133 patients who had been treated with TPE for selected neurological disorders at NGHA in Jeddah, Saudi Arabia, between August 2015 and December 2022. A non-probability consecutive sampling technique was used to include patients who met the inclusion and exclusion criteria.

### 2.3 Inclusion and exclusion criteria

#### 2.3.1 Inclusion criteria

All patients diagnosed with neurological disorders who underwent TPE at NGHA in Jeddah during the study period.

#### 2.3.2 Exclusion criteria

1. Patients who were treated with TPE in another institution.
2. Patients with incomplete medical records.
3. Patients who underwent TPE for non-neurological indications.

**Figure 1:**
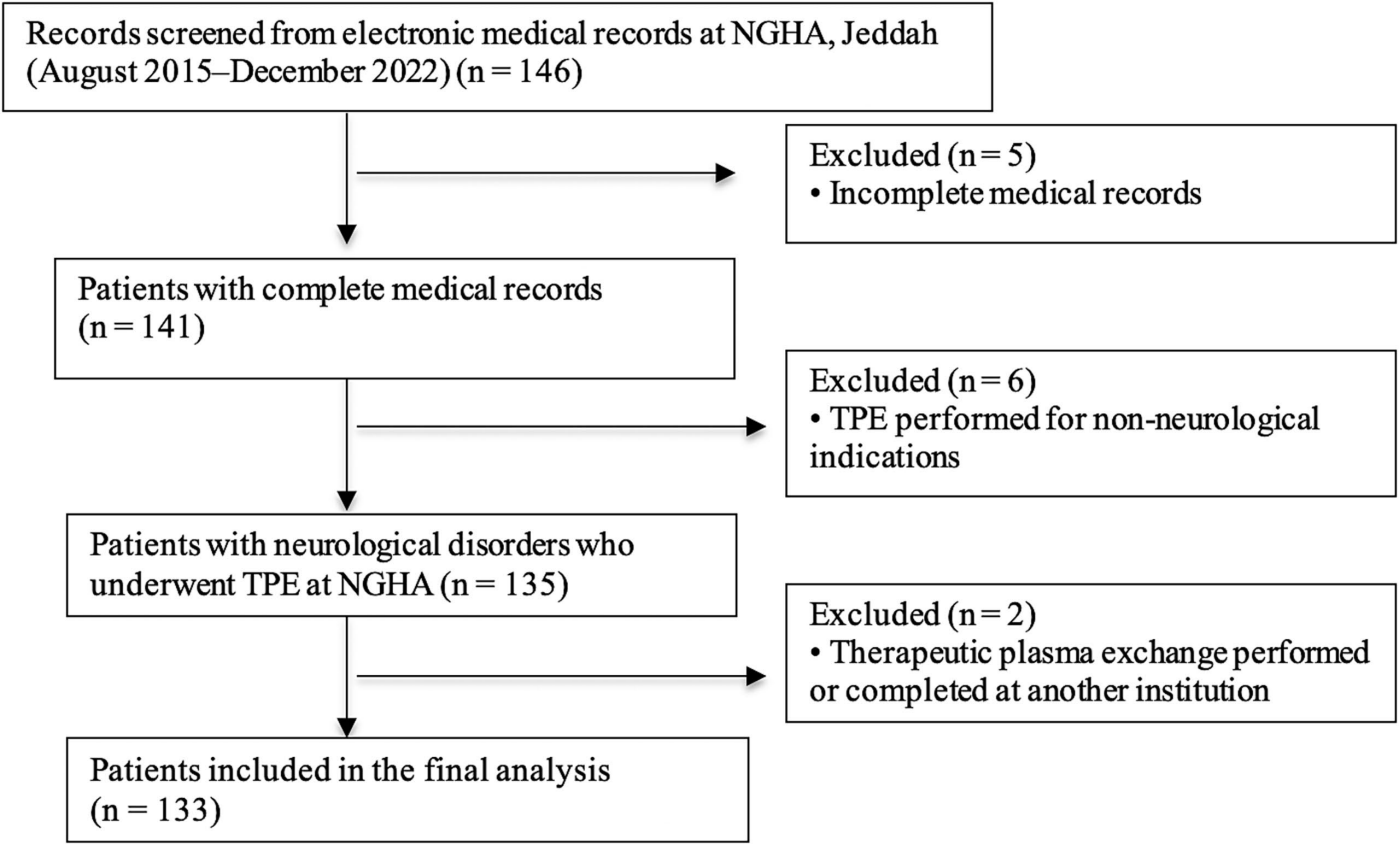
Flow diagram of patient selection. A total of 146 records were screened from electronic medical records at NGHA, Jeddah, between August 2015 and December 2022. Patients were excluded due to incomplete medical records, non-neurological indications for TPE, or TPE performed or completed at another institution, resulting in a final study cohort of 133 patients.

The patient selection process based on these inclusion and exclusion criteria is summarized in Figure 1.

### 2.4 Data collection

Clinical data were retrospectively collected from the Best Care electronic medical records system at NGHA in Jeddah, Saudi Arabia, for all patients with neurological disorders who underwent TPE between August 2015 and December 2022. The included diagnoses were MS, MG, GBS, NMOSD, CIDP, AE, and additional neurological conditions, including amyotrophic lateral sclerosis (ALS), transverse myelitis, cerebellar ataxia, paraneoplastic neurologic syndrome, polymyositis, and central nervous system (CNS) infections. Among patients with myasthenia gravis, TPE was performed for myasthenic crisis and severe exacerbations. No patients received TPE as maintenance therapy.

### 2.5 Measures

Functional status was assessed using the Modified Rankin Scale (mRS), an ordinal scale ranging from 0 to 6, recorded both before and after the TPE course. The mRS scores are defined as follows:

- 0 – No symptoms at all.
- 1 – No significant disability despite symptoms; able to carry out all usual duties and activities.
- 2 – Slight disability; unable to carry out all previous activities but able to look after own affairs without assistance.
- 3 – Moderate disability; requiring some help but able to walk without assistance.
- 4 – Moderately severe disability; unable to walk without assistance and unable to attend to bodily needs without assistance.
- 5 – Severe disability; bedridden, incontinent, and requiring constant nursing care and attention.
- 6 – Dead.

### 2.6 Procedure

TPE involves removing a patient’s blood, separating the plasma, and replacing it with a substitute fluid. According to the NGHA institutional protocol, all patients received a standardized replacement solution during TPE, which aligns with international standards and ASFA guidelines. The replacement fluid typically consisted of 5% albumin, and cryoprecipitate was added for fibrinogen levels <2.5 g/L. A standard course involved 5–7 sessions, with exchange volumes of 1–1.5 plasma volumes per session. In alignment with previous studies, functional outcomes were evaluated 90 days after the final TPE session (16). The analysis was performed at the patient level, and each patient was included only once in the study. For patients who underwent multiple TPE courses during the study period, clinical data from all documented courses were reviewed, and functional outcomes were assessed using the mRS recorded before the first TPE session and 90 days after completion of the final TPE session. Additionally, some patients received both TPE and IVIG. Subgroup analysis was performed to assess differences in outcomes between patients who received TPE alone and those who received combination therapy.

### 2.7 Statistical analysis

Categorical variables were summarized as frequencies and percentages. Continuous variables were assessed for normality; those with a normal distribution were presented as means ± standard deviations (SD). Non-normally distributed continuous data were reported as medians with interquartile ranges (IQR). The Wilcoxon signed-rank test was used to assess changes in mRS scores before and after TPE within each diagnostic group. The Mann–Whitney U test was used to compare the post-treatment mRS scores between patients who received TPE alone and those who received TPE in combination with IVIG. A *p*-value < 0.05 was considered statistically significant. All statistical analyses were performed using IBM SPSS Statistics software, version 29.0 (IBM Corp., Armonk, NY).

## 3 Results

### 3.1 Baseline characteristics

Baseline demographic and comorbidity characteristics are presented in Table 1. There was a slight female predominance (75 patients, 56.4%) compared to 58 male patients (43.6%). The mean age was 47 ± 17.78 years, and the mean BMI was 27.35 ± 7.60 kg/m^2^. Comorbidity assessment revealed a high prevalence of diabetes mellitus (42 patients, 31.6%) and hypertension (39 patients, 29.3%), followed by cardiovascular disease (9 patients, 6.8%) and stroke (7 patients, 5.3%). Neurological comorbidities were notable, with epilepsy reported in 19 patients (14.3%). Chronic kidney disease was present in 14 patients (10.5%), including 5 patients (3.8%) receiving hemodialysis. Additionally, hypothyroidism was reported in 21 patients (15.8%) and systemic lupus erythematosus (SLE) in 6 patients (4.5%). Other reported comorbidities included cancer (24 patients, 18.0%), mental illness (17 patients, 12.8%), and osteoporosis (7 patients, 5.3%). Myasthenic crisis was observed in 2 patients (1.5%).

**Table 1:** Baseline characteristics.

Clinical data		
1. Gender; *n* (%)	Male	58 (43.6%)
	Female	75 (56.4%)
2. Age; (Years) mean ± SD		47.00 ± 17.78
3. Body mass index; (kg/m^2^) mean ± SD		27.35 ± 7.60
4. Diabetes mellitus; *n* (%)	Yes	42 (31.6%)
	No	91 (68.4%)
6. Hypertension; *n* (%)	Yes	39 (29.3%)
	No	94 (70.7%)
7. Dyslipidemia; *n* (%)	Yes	18 (13.5%)
	No	115 (86.5%)
9. Cardiovascular disease; *n* (%)	Yes	9 (6.8%)
	No	124 (93.2%)
10. Stroke; *n* (%)	Yes	7 (5.3%)
	No	126 (94.7%)
11. Epilepsy; *n* (%)	Yes	19 (14.3%)
	No	114 (85.7%)
12. Asthma; *n* (%)	Yes	7 (5.3%)
	No	126 (94.7%)
13. Chronic kidney disease; *n* (%)	Yes	14 (10.5%)
	No	119 (89.5%)
14. Hemodialysis among patients with chronic kidney disease; *n* (%)	Yes	5 (3.8%)
	No	128 (96.2%)
15. Hypothyroidism; *n* (%)	Yes	21 (15.8%)
	No	112 (84.2%)
16. SLE; *n* (%)	Yes	6 (4.5%)
	No	127 (95.5%)
17. Cancers; *n* (%)	Yes	24 (18.0%)
	No	109 (82.0%)
18. Mental Illness; *n* (%)	Yes	17 (12.8%)
	No	116 (87.2%)
19. Osteoporosis *n* (%)	Yes	7 (5.3%)
	No	126 (94.7%)
20. Myasthenic crisis; *n* (%)	Yes	2 (1.5%)
	No	131 (98.5%)

### 3.2 TPE data

Detailed TPE characteristics, including treatment protocol variability and complications, are presented in Table 2. The number of TPE sessions differed across diseases. The mean number of TPE sessions for each patient was recorded as 5.86 ± 3.07, which reflects the variability in treatment intensity required for different neurological disorders and individual patient responses. Femoral vascular access was the most commonly used, accounting for 58 patients (43.6%). In addition, central venous access was used in 41 patients (30.8%), whereas peripheral venous access was used in 4 patients (3.0%). Vascular access type was not documented in 23 patients (17.3%). Overall, 18 of 133 patients (13.5%) experienced minor TPE-related complications, with hypotension occurring in 4 patients (3.0%) and being the most common complication.

**Table 2:** Therapeutic plasma exchange data.

Variable	
Number of TPE sessions; mean ± SD		5.86 ± 3.07
Vascular access methods; *n* (%)	Femoral line	58 (43.6%)
	Central line	41 (30.8%)
	Both peripheral vein and central vein	7 (5.3%)
	Peripheral vein (Hand)	4 (3.0%)
	Undocumented	23 (17.3%)
Complication; *n* (%)	Yes	18 (13.5%)
	No	115 (86.5%)
Type of complication; *n* (%)	Hypotension	4 (3.0%)
	Hemodynamic instability	2 (1.5%)
	Vomiting	2 (1.5%)
	Severe dizziness	2 (1.5%)
	Bleeding	2 (1.5%)
	Allergy/Urticaria	1 (0.8%)
	Burning sensation	1 (0.8%)
	Line block	3 (2.3%)
	Infection	1 (0.8%)
	None	115 (86.5%)

### 3.3 Comparison of functional outcomes between TPE alone and TPE + IVIG

A Mann–Whitney U test was performed to compare the post-treatment mRS scores between patients who received TPE alone and those who received both TPE and IVIG. The test showed no statistically significant difference between the two groups (*U* = 1685.50, *z* = −1.140, *p* = 0.254).

### 3.4 mRS score distribution before and 90 days after TPE in selected neurological disorders

Table 3 presents the distribution of mRS scores before and 90 days after TPE across the different neurological disorders. All patients were symptomatic before treatment. Following TPE, improvement in functional status was observed in several diagnostic groups. In MS, 10 of 24 patients (41.7%) achieved an mRS score of 0, with reductions in moderate disability from 9 to 2 patients and severe disability from 2 to 1 patient. Among patients with MG, 25 of 40 patients (62.5%) achieved an mRS score of 0, and the number of patients with severe disability decreased from 7 to 1, while 3 patients (7.5%) died. In GBS, 12 of 23 patients (52.2%) achieved an mRS score of 0 after TPE, and the number of patients with moderate disability decreased from 8 to 3 (13.0%); one death (4.3%) occurred. For NMOSD, 6 of 9 patients (66.7%) achieved an mRS score of 0, and no patients remained in the moderate disability category after treatment. In CIDP, 5 of 9 patients (55.6%) achieved an mRS score of 0, with no cases of moderate or severe disability and no deaths. Additionally, AE showed functional improvement, with 5 of 10 patients (50.0%) achieving an mRS score of 0 and overall improvement in mild to moderate disability. However, 4 patients (40.0%) remained severely disabled, and 2 patients (20.0%) died. In contrast, outcomes were less favorable among patients with other neurological diseases (*n* = 18). Only 3 patients (16.7%) achieved an mRS score of 0 after TPE, while moderate to severe disability persisted in several cases, and 3 patients (16.7%) died. A detailed diagnosis-specific breakdown of mRS score distribution within the other neurological disorders group is presented in Supplementary Table 1.

**Table 3:** mRS score distribution before and 90 days after TPE in selected neurological disorders.

Diagnosis	*n*	No symptoms* *n* (%)	No significant disability despite symptoms (1) *n* (%)		Slight disability (2) *n* (%)		Moderate disability (3) *n* (%)		Moderately severe disability (4) *n* (%)		Severe (5) *n* (%)		Death (mRS 6), *n* (%)
		After	Before	After	Before	After	Before	After	Before	After	Before	After	After
MS	24	10 (41.7%)	1 (4.2%)	10 (41.7%)	9 (37.5%)	0 (0.0%)	9 (37.5%)	2 (8.3%)	3 (12.5%)	1 (4.2%)	2 (8.3%)	1 (4.2%)	0 (0.0%)
MG	40	25 (62.5%)	6 (15.0%)	8 (20.0%)	15 (37.5%)	1 (2.5%)	11 (27.5%)	1 (2.5%)	1 (2.5%)	1 (2.5%)	7 (17.5%)	1 (2.5%)	3 (7.5%)
GBS	23	12 (52.2%)	2 (8.7%)	3 (13.0%)	6 (26.1%)	3 (13.0%)	8 (34. 8%)	2 (8.7%)	5 (21.7%)	2 (8.7%)	2 (8.7%)	0 (0.0%)	1 (4.3%)
NMOSD	9	6 (66.7%)	0 (0.0%)	2 (22.2%)	4 (44.4%)	0 (0.0%)	4 (44.4%)	0 (0.0%)	1 (11.1%)	1 (11.1%)	0 (0.0%)	0 (0.0%)	0 (0.0%)
CIDP	9	5 (55.6%)	0 (0.0%)	1 (11.1%)	4 (44.4%)	3 (33.3%)	5 (55.6%)	0 (0.0%)	0 (0.0%)	0 (0.0%)	0 (0.0%)	0 (0.0%)	0 (0.0%)
AE	10	5 (50.0%)	0 (0.0%)	1 (10.0%)	3 (30.0%)	0 (0.0%)	3 (30.0%)	1 (10.0%)	0 (0.0%)	0 (0.0%)	4 (40.0%)	1 (10.0%)	2 (20.0%)
Other neurological diseases*	18	3 (16.7%)	0 (0.0%)	2 (11.1%)	3 (16.7%)	1 (5.6%)	6 (33.3%)	3 (16.7%)	4 (22.2%)	3 (16.7%)	5 (27.8%)	3 (16.7%)	3 (16.7%)

mRS, modified Rankin Scale; TPE, therapeutic plasma exchange; MS, multiple sclerosis; MG, myasthenia gravis; GBS, Guillain–Barré syndrome; NMOSD, neuromyelitis optica spectrum disorder; CIDP, chronic inflammatory demyelinating polyneuropathy; AE, autoimmune encephalitis.

*Other neurological disorders: included amyotrophic lateral sclerosis, transverse myelitis, cerebellar ataxia, paraneoplastic neurologic syndrome, polymyositis, and central nervous system (CNS) infections.

*All patients were symptomatic before treatment.

### 3.5 Changes in mRS scores before and 90 days after TPE in selected neurological disorders

The Wilcoxon signed-rank test revealed statistically significant reductions in mRS scores following TPE in patients with MS, MG, GBS, NMOSD, CIDP, and AE, as shown in Table 4. Median mRS scores decreased from 3 to 1 in MS, from 2 to 0 in MG, and from 3 to 0 in GBS, all with *p* < 0.001. Similarly, median mRS scores decreased in NMOSD from 3 to 0 (*p* = 0.008), in CIDP from 3 to 0 (*p* = 0.007), and in AE from 3 to 0.5 (*p* = 0.018), although the interquartile range widened, indicating variability in treatment response. In contrast, no statistically significant change was observed in patients with other neurological diseases, with the median mRS remaining at 3.5 before and 90 days after TPE (*p* = 0.234). Overall, statistically significant reductions in mRS scores were observed following TPE in most diagnostic categories.

**Table 4:** Changes in mRS scores before and 90 days after TPE in selected neurological disorders.

Diagnosis	Median		Interquartile range		Z-score	*p*-values
	Pre-TPE	Post-TPE	Pre-TPE	Post-TPE		
Multiple sclerosis	3	1	1	1	−4.242	<0.001
Myasthenia gravis	2	0	1	1	−5.158	<0.001
Guillain–Barré syndrome	3	0	2	2	−3.952	<0.001
NMOSD	3	0	1	1	−2.640	0.008
CIDP	3	0	1	2	−2.701	0.007
Autoimmune encephalitis	3	0.50	3	5	−2.356	0.018
Other neurological diseases^*^	3.5	3.5	2	4	−1.190	0.234

mRS, modified Rankin Scale; TPE, therapeutic plasma exchange; NMOSD, neuromyelitis optica spectrum disorder; CIDP, chronic inflammatory demyelinating polyneuropathy.

^*^Other neurological disorders: included amyotrophic lateral sclerosis, transverse myelitis, cerebellar ataxia, paraneoplastic neurologic syndrome, polymyositis, and central nervous system (CNS) infections.

### 3.6 Diagnostic-specific clinical characteristics, treatment patterns, and mortality

Table 5 presents diagnostic-specific clinical characteristics, treatment patterns, mortality, and TPE sessions. The largest diagnostic group was MG (*n* = 40), followed by MS (*n* = 24), GBS (*n* = 23), other neurological diseases (*n* = 18), AE (*n* = 10), NMOSD (*n* = 9), and CIDP (*n* = 9). Female predominance was observed in MS, MG, GBS, NMOSD, and AE, whereas male predominance was observed in CIDP and other neurological diseases. Mean age ranged from 38.96 ± 12.90 years in MS to 53.44 ± 12.01 years in NMOSD. TPE alone was used in most diagnostic groups, except for GBS, where TPE combined with IVIG was more frequent (12 patients, 52.2%). Mortality was reported in MG (3 patients, 7.5%), GBS (1 patient, 4.3%), AE (2 patients, 20.0%), and other neurological diseases (3 patients, 16.7%). The mean number of TPE sessions ranged from 3.80 ± 1.55 in AE to 6.97 ± 4.66 in MG.

**Table 5:** Diagnostic-specific clinical characteristics, treatment patterns, and mortality.

Variables	*n*	Gender *n* (%)		Age (years), mean ± SD	TPE alone *n* (%)	TPE + IVIG *n* (%)	Mortality *n* (%)	TPE sessions, mean ± SD
		Male	Female					
Multiple sclerosis	24	9 (37.5%)	15 (62.5%)	38.96 ± 12.90	19 (79.2%)	5 (20.8%)	0 (0.0%)	5.00 ± 1.79
Myasthenia gravis	40	17 (42.5%)	23 (57.5%)	51.22 ± 17.95	31 (77.5%)	9 (22.5%)	3 (7.5%)	6.97 ± 4.66
Guillain–Barré syndrome	23	9 (39.1%)	14 (60.9%)	47.09 ± 23.86	11 (47.8%)	12 (52.2%)	1 (4.3%)	5.57 ± 1.31
NMOSD	9	1 (11.1%)	8 (88.9%)	53.44 ± 12.01	6 (66.7%)	3 (33.3%)	0 (0.0%)	5.78 ± 1.56
CIDP	9	8 (88.9%)	1 (11.1%)	45.44 ± 12.07	5 (55.6%)	4 (44.4%)	0 (0.0%)	6.44 ± 2.01
Autoimmune encephalitis	10	3 (30.0%)	7 (70.0%)	43.80 ± 25.01	8 (80.0%)	2 (20.0%)	2 (20.0%)	3.80 ± 1.55
Other neurological diseases	18	11 (61.1%)	7 (38.9%)	47.56 ± 11.08	11 (61.1%)	7 (38.9%)	3 (16.7%)	5.78 ± 2.34

TPE, therapeutic plasma exchange; NMOSD, neuromyelitis optica spectrum disorder; CIDP, chronic inflammatory demyelinating polyneuropathy.

*Other neurological diseases: including Amyotrophic Lateral Sclerosis, Transverse Myelitis, Cerebellar Ataxia, Paraneoplastic Neurologic Syndrome, polymyositis, and Central Nervous System (CNS) infections.

### 3.7 Patient demographics and comorbidities in mortality cases following TPE

Among the 133 patients included in the study, 9 patients (6.8%) died during the 90-day follow-up period after TPE. The diagnoses among deceased patients included MG in 3 patients (33.3%), AE in 2 patients (22.2%), transverse myelitis in 2 patients (22.2%), GBS in 1 patient (11.1%), and ALS in 1 patient (11.1%). Most deceased patients were female (7 patients, 77.8%), with a mean age of 66.7 ± 17 years and a mean BMI of 27.8 ± 2.8 kg/m^2^. Frequently reported comorbidities included hypertension (6 patients, 66.7%), diabetes mellitus (5 patients, 55.6%), cancer (5 patients, 55.6%), chronic kidney disease (4 patients, 44.4%), and cardiovascular disease (4 patients, 44.4%). Less common comorbidities included osteoporosis (2 patients, 22.2%), asthma, mental illness, and hemodialysis (1 patient each, 11.1%). Myasthenic crisis was documented in 1 patient (11.1%). Additionally, 3 of the deceased patients (33.3%) had received both TPE and IVIG (Table 6).

**Table 6:** Patient demographics and comorbidities in mortality cases following TPE.

Variables		
Diagnosis; *n* (%)	Myasthenia gravis	3 (33.3%)
	Guillain–Barré syndrome	1 (11.1%)
	Autoimmune encephalitis	2 (22.2%)
	Other neurological diseases (Transverse myelitis)	2 (22.2%)
	Other neurological diseases (Amyotrophic lateral sclerosis)	1 (11.1%)
Gender; *n* (%)	Female	7 (77.8%)
	Male	2 (22.2%)
Age; (Years) mean ± SD	66.67 ± 17	
Body mass index; (kg/m^2^) mean ± SD	27.82 ± 2.8	
Diabetes mellitus; *n* (%)	Yes	5 (55.6%)
	No	4 (44.4%)
Hypertension; *n* (%)	Yes	6 (66.7%)
	No	3 (33.3%)
Dyslipidemia; *n* (%)	Yes	3 (33.3%)
	No	6 (66.7%)
Cardiovascular disease; *n* (%)	Yes	4 (44.4%)
	No	5 (55.6%)
Asthma; *n* (%)	Yes	1 (11.1%)
	No	8 (88.9%)
Chronic kidney disease; *n* (%)	Yes	4 (44.4%)
	No	5 (55.6%)
Hemodialysis; *n* (%)	Yes	1 (11.1%)
	No	8 (88.9%)
Cancer; *n* (%)	Yes	5 (55.6%)
	No	4 (44.4%)
Mental illness; *n* (%)	Yes	1 (11.1%)
	No	8 (88.9%)
Osteoporosis; *n* (%)	Yes	2 (22.2%)
	No	7 (77.8%)
Myasthenic crisis; *n* (%)	Yes	1 (11.1%)
	No	8 (88.9%)
Intravenous immunoglobulin (IVIG); *n* (%)	Yes	3 (33.3%)
	No	6 (66.7%)

## 4 Discussion

### 4.1 Summary of evidence

This study evaluated the association between TPE and functional outcomes in neurological disorders. Overall, TPE was associated with improved functional outcomes in several neurological disorders, findings that are broadly consistent with current guideline recommendations. The majority of patients in the study who underwent TPE had autoimmune neurological disorders (118 patients), while only 15 patients were treated for non-autoimmune neurological conditions. Overall, slightly more than half of the patients were female (56.4%), in accordance with the literature indicating autoimmune disorders are more prevalent in women, likely due to hormonal and genetic reasons. Ortona et al. (17) discuss the role of biological differences between males and females in the immunological mechanisms of autoimmune disorders and their various autoimmune incidences. Moreover, our cohort also frequently had co-occurring autoimmune disorders, with hypothyroidism occurring in 15.8% and SLE in 4.5% of patients. This was consistent with the findings of Abend et al. (18), who reported that 34% of the patients with an autoimmune disorder had at least one additional autoimmune disorder.

Moreover, the association between TPE and functional outcomes varied across different neurological disorders, as assessed by the mRS. The study observed statistically significant reductions in mRS score following TPE in patients with MS (*p* < 0.001), MG (*p* < 0.001), GBS (*p* < 0.001), NMOSD (*p* = 0.008), CIDP (*p* = 0.007), and AE (*p* = 0.018), indicating lower disability scores after treatment. The mRS was selected as the primary outcome measure because it was consistently documented across all diagnostic groups and available retrospectively for the entire cohort. However, given the heterogeneous nature of the study population, the mRS may not fully capture disease-specific clinical changes. Disease-specific outcome measures, such as the Expanded Disability Status Scale (EDSS) for MS and NMOSD, the Myasthenia Gravis Activities of Daily Living (MG-ADL) scale and Myasthenia Gravis Foundation of America (MGFA) Clinical Classification for MG, and the Guillain–Barré Syndrome Disability Scale, were not uniformly available in the medical records and therefore could not be evaluated. Consequently, some disease-specific improvements may not have been fully reflected in the outcome assessment. These findings are broadly consistent with the American Society for Apheresis (ASFA) guideline recommendations. TPE is classified as a Category I indication for GBS, short-term management of MG, CIDP, and NMOSD during acute relapses, with grades of recommendation ranging from 1A to 1C (11). MS is classified as a Category II indication, which is also consistent with the observed reductions in mRS scores in our cohort. In addition, antibody-mediated autoimmune encephalitis, particularly anti-N-methyl-D-aspartate receptor (anti-NMDAR) encephalitis, is classified as a Category I indication, whereas other autoimmune encephalitis subtypes fall into Categories II or III (19).

In contrast, patients with other neurological disorders, including transverse myelitis, ALS, paraneoplastic neurological syndromes, and autoimmune cerebellar ataxia, did not demonstrate a significant change in mRS scores following TPE (*p* = 0.234). These disorders are generally classified within ASFA Categories III or IV, where the role of TPE remains uncertain or is recommended only on a case-by-case basis. Overall, our findings are consistent with current ASFA classifications and suggest that TPE may be associated with favorable functional outcomes in selected neurological disorders, while highlighting the importance of individualized clinical judgment in conditions with limited or conflicting evidence (11).

Comorbidities may have influenced treatment outcomes. Among patients who died during the 90-day follow-up period, conditions such as hypertension, diabetes mellitus, chronic kidney disease, malignancy, and cardiovascular disease were prevalent. This finding is consistent with the findings of Gertz et al., which highlighted that while plasmapheresis can be used in certain malignancy-associated neurological conditions, outcomes are often poor unless the underlying cancer is simultaneously treated (20). Although no TPE-related deaths were identified based on the available clinical documentation in this cohort, the prevalence of multiple chronic comorbidities among deceased patients suggests that these conditions may have contributed to poorer outcomes. However, the study was not designed to determine independent predictors of mortality. In addition, one-third of the deceased patients had received both TPE and IVIG; however, the limited number of deaths and potential confounding by indication preclude conclusions regarding the effect of combination therapy in this subgroup. These findings highlight the importance of careful patient selection and individualized management, especially in older patients with multiple comorbidities, and the need to consider integrated management and individualized risk.

### 4.2 Limitations

This study has several limitations. The retrospective design and descriptive analysis limit causal inference. In addition, the use of retrospective data from patients treated with TPE for neurological disorders at NGHA in Jeddah, along with the absence of random sampling, limits the generalizability of these findings to broader populations. The heterogeneous study population and small sample sizes in some diagnostic subgroups, particularly NMOSD, CIDP, and autoimmune encephalitis, also limit the power for subgroup analyses and may have affected the robustness of the outcomes. Functional outcomes were assessed solely using the mRS, which may not fully capture disease-specific clinical changes. Disease-specific outcome measures, such as the EDSS, MG-ADL scale, MGFA clinical classification, and GBS Disability Scale, were not uniformly available and therefore could not be analyzed. As a result, some disease-specific clinical changes may not have been fully captured. Concomitant therapies, including IVIG and other disease-specific treatments, may have influenced outcomes. Although subgroup analysis did not show a significant difference in functional outcomes between TPE alone and TPE combined with IVIG, the potential for treatment-related confounding remains. The study also relied on data collected from medical records, which may be subject to measurement error due to incomplete or inaccurate documentation. Detailed information regarding prior corticosteroid use and immunosuppressive therapies was not consistently available; therefore, these unmeasured therapies may have influenced post-TPE outcomes. Additionally, the impact of TPE-related adverse events on treatment interruption, vascular access removal, or protocol modification was not consistently documented. Finally, missing vascular access data in some patients and the inability to adjust for confounding in regression analysis may have introduced bias, and residual confounding remains.

## 5 Conclusion

This study suggests that TPE could be associated with improved functional outcomes in several selected neurological disorders, including MS, MG, GBS, NMOSD, CIDP, and AE, which is broadly consistent with current ASFA guideline recommendations. In contrast, no statistically significant improvement was observed in other neurological conditions, which may reflect the uncertain or less established role of TPE in these disorders. Due to the retrospective design, disease heterogeneity, and potential treatment-related confounding, causal inferences cannot be made. Since the analyses were primarily descriptive, this study should be considered exploratory; however, it lays the foundation for future studies using multivariable and stratified analyses. Nevertheless, these findings provide real-world evidence regarding the association between TPE and functional outcomes in selected neurological disorders and highlight the need for prospective multicenter studies to further evaluate its role and optimize patient selection.

## Data Availability

The raw data supporting the conclusions of this article can be made available by the corresponding author upon request, in accordance with applicable ethical and confidentiality requirements.
