# Supplementary material for: Association of therapeutic plasma exchange with functional outcomes in selected neurological disorders: a retrospective cohort study from a single tertiary center in Saudi Arabia
Source: Front Neurol. 2026 Jul 21;17:1876331. doi: 10.3389/fneur.2026.1876331 (PMC13433597; doi:10.3389/fneur.2026.1876331)
Supplement: Supplementary file 1 [file Table_1.DOCX]

**Supplement Table 1. modified Rankin Scale (mRS) Distribution Before and 90 Days After therapeutic plasma exchange (TPE) in Other Neurological Disorders**

| **Diagnosis** | **n** | **No Symptoms * *n (%)*** | **No significant disability despite symptoms (1) *n (%)*** | | **Slight disability (2) *n (%)*** | | **Moderate disability (3) *n (%)*** | | **Moderately severe disability *n (%)*** | | **Severe (5) *n (%)*** | | **Dead (6)* *n (%)*** |
| --- | --- | --- | --- | --- | --- | --- | --- | --- | --- | --- | --- | --- | --- |
|  |  | After | Before | After | Before | After | Before | After | Before | After | Before | After | After |
| ALS | 6 | 0 (0.0%) | 0 (0.0%) | 0 (0.0%) | 0 (0.0%) | 0 (0.0%) | 1 (16.7%) | 0 (0.0%) | 3 (50.0%) | 3 (50.0 %) | 2 (33.3%) | 2 (33.3%) | 1 (16.7%) |
| Transverse myelitis | 5 | 0 (0.0%) | 0 (0.0%) | 0 (0.0%) | 0 (0.0%) | 0 (0.0%) | 2 (40.0%) | 1 (20.0%) | 0 (0.0%) | 1 (4.2%) | 3 (60.0%) | 1 (20.0%) | 2 (40.0%) |
| Cerebellar ataxia | 1 | 0 (0.0%) | 0 (0.0%) | 0 (0.0%) | 1(100.0%) | 1(100.0%) | 0 (0.0%) | 0 (0.0%) | 0 (0.0%) | 0 (0.0%) | 0 (0.0%) | 0 (0.0%) | 0 (0.0%) |
| CNS infection | 1 | 0 (0.0%) | 0 (0.0%) | 0 (0.0%) | 0 (0.0%) | 0 (0.0%) | 1 (100.0%) | 1 (100.0%) | 0 (0.0%) | 0 (0.0%) | 0 (0.0%) | 0 (0.0%) | 0 (0.0%) |
| Paraneoplastic syndrome | 3 | 2 (66.7%) | 0 (0.0%) | 1 (33.3%) | 1 (33.3%) | 0 (0.0%) | 2 (66.7%) | 0 (0.0%) | 0 (0.0%) | 0 (0.0%) | 0 (0.0%) | 0 (0.0%) | 0 (0.0%) |
| Polymyositis | 2 | 1 (50.0%) | 0 (0.0%) | 1 (50.0%) | 1 (50.0%) | 0 (0.0%) | 1 (50.0%) | 0 (0.0%) | 0 (0.0%) | 0 (0.0%) | 0 (0.0%) | 0 (0.0%) | 0 (0.0%) |

**ALS:** Amyotrophic Lateral Sclerosis, **(CNS) infection:** Central Nervous System infection
